# Adaptive Recognition of Bioacoustic Signals in Smart Aquaculture Engineering Based on r-Sigmoid and Higher-Order Cumulants

**DOI:** 10.3390/s22062277

**Published:** 2022-03-15

**Authors:** Tianyu Cao, Xiaoqun Zhao, Yichen Yang, Caiyun Zhu, Zhongwei Xu

**Affiliations:** Department of Information and Communication Engineering, College of Electronic and Information Engineering, Tongji University, Jiading District, Shanghai 201804, China; zhao_xiaoqun@tongji.edu.cn (X.Z.); yicen@tongji.edu.cn (Y.Y.); 2130697@tongji.edu.cn (C.Z.); 2180135@tongji.edu.cn (Z.X.)

**Keywords:** underwater acoustic signals, SRC, HOC, recognition-sigmoid function

## Abstract

In recent years, interest in aquaculture acoustic signal has risen since the development of precision agriculture technology. Underwater acoustic signals are known to be noisy, especially as they are inevitably mixed with a large amount of environmental background noise, causing severe interference in the extraction of signal features and the revelation of internal laws. Furthermore, interference adds a considerable burden on the transmission, storage, and processing of data. A signal recognition curve (SRC) algorithm is proposed based on higher-order cumulants (HOC) and a recognition-sigmoid function for feature extraction of target signals. The signal data of interest can be accurately identified using the SRC. The analysis and verification of the algorithm are carried out in this study. The results show that when the SNR is greater than 7 dB, the SRC algorithm is effective, and the performance improvement is maximized when the SNR is 11 dB. Furthermore, the SRC algorithm has shown better flexibility and robustness in application.

## 1. Introduction

With the development of new technologies, the aquaculture industry has gradually changed from the traditional labor-intensive model to the smart aquaculture model [1,2,3,4]. Traditional labor-intensive aquaculture mainly depends on the experience of farmers, with high labor costs and low work efficiency [5]. However, smart aquaculture can use sensors (i.e., hydrophones, cameras, thermometers, etc.) to monitor changes in aquatic status (e.g., resting, feeding, etc.) and living environment (e.g., water temperature, dissolved oxygen, PH value, etc.) to automatically adjust the aquaculture operation plan, which, in turn, significantly improves production and reduces labor costs [6,7,8]. Currently, one of the major difficulties in monitoring the status of aquatic animals is that traditional monitoring using cameras in water is very ineffective due to turbid water and other reasons, whereas hydrophones can sense underwater acoustic signals, which has inspired us to identify the status of aquatic animals by the sounds they make.

Bioacoustic hydrophones are the most basic information sensing equipment in smart aquaculture engineering. Generally, aquatic animals make different sounds under different conditions [9]. Bioacoustic hydrophones can be used to monitor relevant physiology, behavior, and other conditions [9,10,11,12]. Therefore, hydrophones play a vital role in the development of aquaculture [13,14,15]. Furthermore, acoustic monitoring results can reflect animal health and environmental changes [15]. Accordingly, this information can be fed back to the Smart Aquaculture Management System [15]. The healthy breeding of aquatic organisms requires long-term monitoring with hydrophones [14,16]. However, efficient expression and storage of the original monitoring data have become important issues in aquaculture engineering [17,18].

**Figure 1:**
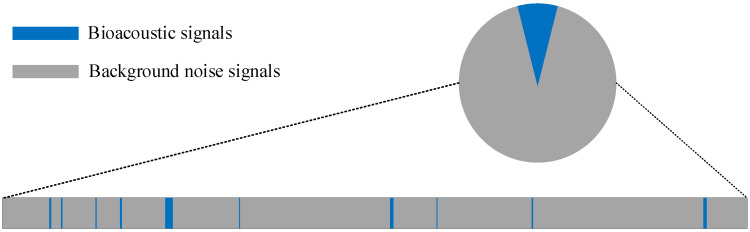
Diagrammatic sketch of distribution and proportion of bioacoustic signals and background noise signals in aquaculture.

Long-term monitoring data show that the proportion of acoustic signals from aquatic animals is very small, and most of the data are from environmental background noises [19]. The effective vocalizations of aquatic animals are primarily short, sparse, non-stationary signals. Figure 1 shows a short-term random, sparse distribution. The blue area represents bioacoustic signals, and the gray area represents environmental background noise signals, which occupy most of the monitoring time and storage space. Therefore, the majority of the stored data are redundant environmental background noises, making it challenging to identify the signal data through time [19,20]. It is also difficult to accurately locate and process the signal on the time axis during data post-processing. With the increase in the amount of monitoring signal data, in order to solve the problem of effective information classification and extraction in long-term monitoring data, dynamic data mining technology [21,22], signal data pretreatment technology [23], deep neural networks [12], and other methods are proposed. However, it cannot be applied to the problems of target signal screening and recognition in aquaculture engineering.

In the actual aquaculture environment, the statistical characteristics of the underwater acoustic observation signal are usually a Gaussian mixture [24]. There are limitations in the first-order and second-order statistical signal processing of the signal characteristics, and therefore, their statistical characteristics cannot be fully represented. Meanwhile, higher-order statistical signal processing contains higher-order characteristic information of the signal that can suppress the additive noise of the unknown power spectrum, such as higher-order moments, higher-order cumulants, higher-order spectra, etc. [25]. Thus, it has unique advantages for noise suppression in signal detection. In this paper, a signal recognition curve (SRC) algorithm based on the combination of the HOC and r-sigmoid function is proposed according to the actual monitoring requirements of *Penaeus vannamei* and the actual acoustic field environment in aquaculture. The SRC method enables the hydrophone to identify a specified bioacoustic signal in the water while actively suppressing or ignoring the irrelevant background noise signals. This algorithm can directly transmit a small amount of effective monitoring information, rather than a large amount of ineffective monitoring data.

The structure of the rest of the paper is organized as follows. The theoretical derivation and analysis of the HOC and r-sigmoid functions are provided in Section 2. The algorithm performance through simulation signal processing is analyzed, and the algorithm’s effectiveness through further analysis of the application case is verified. The experimental results are summarized, and the research content is discussed in Section 3. Finally, the research of this paper is summarized, and the application prospects of the future are presented in Section 4.

## 2. Algorithm Theory Derivation

According to the demand for smart aquaculture applications, this study uses higher-order statistics for underwater acoustic signals (such as target detection). Higher-order statistics can be applied to non-stationary sparsity structures of a detected signal time series, and various types of additive noises can also be effectively suppressed with their use. The higher-order statistics of the signal, which can be calculated and obtained from the signal data samples, can provide the probability density function in many aspects.

Assuming that the probability density function of the acoustic signal s(x) of *Penaeus vannamei* in aquaculture is p(x), the moment generation function is the Fourier transform of the probability density function, as shown in the following equation:

(1) $$\varphi (w)={\int p(x)e}^{i(wx)}dx$$

Then, the moment generating function of the multivariate probability density function $p_{x}(x)$ of the n-dimensional zero-mean vector ***x*** can be generalized as:

(2) $$\varphi_{x}(w)={\int p_{x}\left(x\right)e}^{i(wx)}dx$$

The cumulant generator function is defined as the log of the moment generator function:

(3) $$\phi_{}(w)=\log(\varphi_{x}(w))$$

The Taylor series expansion is then performed on Equation (3):

(4) $$\phi (w)={\sum_{l_{1}+l_{2}+\ldots l_{N}=0}^{\infty }\frac{i^{\left(l_{1}+l_{2}\ldots +l_{N}\right)}}{l_{1}!+l_{2}!\ldots l_{N}!}}^{}k_{x}^{\left(l_{1},l_{2},\ldots l_{N}\right)}w_{1}^{l_{1}}w_{2}^{l_{2}}\ldots w_{N}^{l_{N}}$$

According to Taylor series theory, the joint cumulative quantity of the $l=l_{1}+l_{2}+\ldots +l_{N}$ order is:

(5) $$k_{x}^{\left(l_{1},l_{2},\ldots ,l_{N}\right)}={\left(-i\right)}^{l}\frac{\partial^{l}\log(\varphi_{x}(w))}{\partial w_{1}^{l_{1}}\partial w_{2}^{l_{2}}\ldots \partial w_{N}^{l_{N}}}{|}_{w_{1}=w_{2}\ldots w_{N}=0}$$

Then, the third-order cumulant can be calculated as:

(6) $$k_{x}{}^{\left(3\right)}={\left(-i\right)}^{3}\frac{d^{3}\phi (w)}{dw^{3}}{|}_{w=0}{}_{}=m_{3}-3m_{1}m_{2}+2m_{1}^{3}$$

Here, $m_{k}$ is the K-order moment, as follows:

(7) $$m_{k}=E[x^{k}]=\int x^{k}p(x)dx\;\;\;k=1,2,3$$

**Figure 2:**
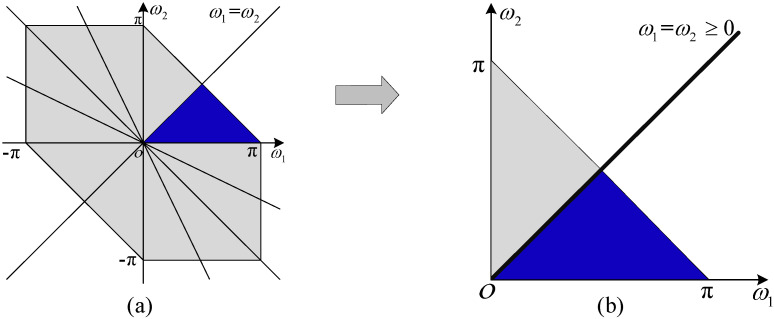
Symmetrical areas of bispectrum. (**a**) Twelve symmetric areas of the bispectrum. (**b**) One of the effective areas of the bispectrum.

Therefore, the statistical properties of the random variables in the time domain space can be fully described by higher-order moments and higher-order cumulants. Meanwhile, the HOC spectrum can be used to describe the frequency domain space. In this paper, the third-order spectrum (bispectrum) was used to describe the frequency domain space, as follows:

(8) $$A_{x}{}^{\left(3\right)}(\omega_{1},\omega_{2})=\left|A_{x}{}^{\left(3\right)}(\omega_{1},\omega_{2})\right|e^{j\phi_{A}(\omega_{1},\omega_{2})}$$

where $\left|A_{x}{}^{\left(3\right)}(\omega_{1},\omega_{2})\right|$ represents the amplitude of the bispectrum, and $j\phi_{A}^{}(\omega_{1},\omega_{2})$ represents the phase of the bispectrum. According to the symmetry of the bispectrum, there are 12 symmetrical areas, as shown in Figure 2. Thus, the bispectrum can be completely described by the blue area ($\omega_{1}-\omega_{2}\geq 0$, $\omega_{1}+\omega_{2}\leq \pi$, $\omega_{2}\geq 0$) in Figure 2, where the spectrum can be described as $\omega_{1}=\omega_{2}\geq 0$.

In this study, the environmental background noise and the target signal can be distinguished from the time and frequency domains by calculating the HOC and bispectrum, respectively. In order for the bioacoustic hydrophone to recognize the target signal, the sigmoid function is introduced, and the r-sigmoid function is constructed with the following expression:

(9) $$\begin{array}{l} \mathrm{Sigmoid}(x)=\frac{1}{1+e^{-x}}\\ \Rightarrow r-\mathrm{Sigmoid}(x)=\frac{1}{1+e^{-\lambda (x-\xi )}} \end{array}$$

The unknown signal s(x) is comprehensively processed by combining the HOC and the recognition function. In this way, Equation (10) can retain the noise suppression performance of the HOC while having the ability to identify functions as follows:

(10) $$\begin{array}{ll} s\prime (x) & =s(x)k{\left(x\right)}^{\left(3\right)}{}_{}r-\mathrm{Sigmoid}(x)\\ & =s(x)\times {\left(-i\right)}^{3}\frac{d^{3}\phi (w)}{dw^{3}}{|}_{w=0}\times \frac{1}{1+e^{-\lambda (x-\xi )}} \end{array}$$

**Figure 3:**
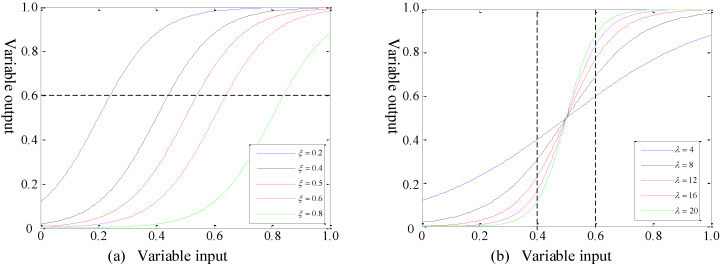
Recognition function curves. (**a**) The influence of different values of recognition coefficient *ξ* on the curve, when *λ* = 12. (**b**) The influence of different values of recognition coefficient *λ* on the curve, when *ξ* = 0.5.

According to Equation (10), the performance of the recognition function of the HOC was determined by the recognition coefficients *λ* and *ξ*. We control a single variable and discuss the influence of *λ* and *ξ* on the recognition performance separately. The recognition function curves are shown in Figure 3.

First, we discuss the influence of different values of *ξ* on the curve under the condition that *λ* = 12 is fixed, as shown in Figure 3a. Then, with *ξ* = 0.5 fixed, the influence of different values of *λ* on the curve is discussed, as shown in Figure 3b. Therefore, the recognition performance of the hydrophone bioacoustics can be jointly determined by the settings of the recognition coefficients *λ* and *ξ* in the r-sigmoid function. Combined with the HOC and recognition function, the hydrophone can recognize and classify animal acoustic signals in the aquaculture environment. In this paper, the acoustic signals processing and estimation method is illustrated in Algorithm 1.

**Algorithm 1:** The Signal Recognition Curve (SRC) Algorithm.1:**Parameter setting:** Signal frame length.2: Set the recognition coefficients *λ* and *ξ*.3: Set the recognition threshold Thr.4:**Input:** Underwater observation signal *s*(*x*)5:**while** Signal length > Signal frame length **do**6: Signal framing **→** *s_i_*(*x*)7: **for** each frame *s_i_*(*x*) in *s*(*x*) **do**8:  Calculate the higher-order cumulant **→** *k_i_*(*x*)^3^9:  Calculate the recognition-sigmoid function → r-Sigmoid*_i_*(*x*)10:  Calculate the signal recognition curve → SRC*_i_*(*x*)11:  **if** SRC*_i_*(*x*) > Thr **then**
   Target Signal Recognition → Detection*_i_*(*x*) = 112:  **else**
   Target Signal Recognition → Detection*_i_*(*x*) = 013:   Short-term noise suppression → *s_i_*′(*x*) = SRC*_i_*(*x*) · *s_i_*(*x*)  **end while**14:**Output:** The SRC of the observation signal → SRC(*x*)
 Target signal detection area → Detection(*x*)
 The signal after the SRC algorithm processing → *s*′(*x*)

## 3. Experiments and Analysis

This section primarily includes two parts: simulation experiments and verification experiments. Simulation experiments can provide more valuable references for verification experiments.

### 3.1. Design of Simulation Signal

**Figure 4:**
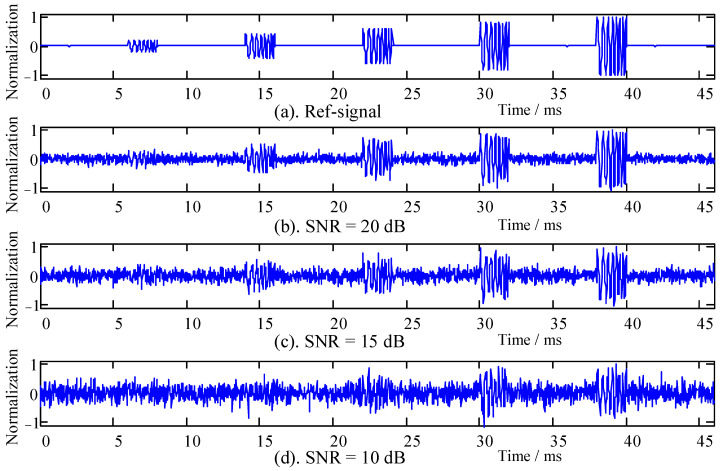
Simulated signals with different SNRs. (**a**) Simulated reference signal. (**b**–**d**) The background noise with different SNRs of 20, 15, and 10 dB, respectively.

In this study, the acoustic signal of *Penaeus vannamei* is designed to simulate the smart aquaculture environment. The acoustic signal was short-term, random, and sparse, as referred to in the literature [26]. Five different LFM pulse signals were designed to simulate the acoustic signals in some scenes. The signal’s sampling frequency was 48 kHz, the central frequency of the signals was 7 kHz, the signal bandwidth was 2 kHz, and the pulse time was 2 ms, with amplitudes from small to large, as shown in Figure 4. The normalized amplitude ratios of the signals were 1:2:3:4:5, respectively. The simulation reference signal was composed of five pulse signals with a 6 ms interval to simulate the actual sounding phenomenon and environmental background noise, as shown in Figure 4.

Figure 4 shows that the target signal interfered with different degrees in the background noise with different SNRs. The following two points are shown in Figure 4. (1) When the signal amplitude is the same, the larger the background noise power, the smaller the SNR, and the more severe the signal is overwhelmed by interference. (2) When the background noise power is fixed, the smaller the signal amplitude, and the more severe the signal is submerged by the interference. The processing of the recognition algorithm in this paper was based on the simulation signal in Figure 4, in which three groups of control experiments with different SNRs are provided.

### 3.2. Noise Suppression Performance of the HOC

**Figure 5:**
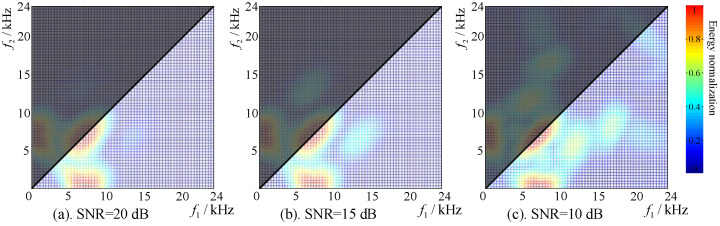
Comparison of higher-order spectrum under different SNRs.

The environmental background noise in the aquaculture environment is usually additive Gaussian mixture noise. The ability of the signal processing algorithm to suppress background noise is a key factor. According to Equations (6) and (8), the HOC spectrum (bispectrum) of simulation signals with different SNRs were analyzed as shown in Figure 5a–c, corresponding to the bispectrum with SNRs of 20, 15, and 10 dB, respectively.

The signals with different SNRs in Figure 5 were comparatively analyzed. When the SNR was low (SNR = 15 dB and SNR = 10 dB), there were interference components in the bispectrum space, as in Figure 5b,c. With the increase in SNR, the interference in the bispectrum decreased, as in Figure 5a. However, this paper mainly focuses on the influence of SNR variation on frequency domain space, so the difference comparison in the frequency domain space can be performed through the diagonal ($f_{1}=f_{2}\geq 0$). It can be seen that additive Gaussian noise had little effect on the frequency domain of the target signal, with the signal frequency spectrum ranging from 6 ± 0.5 kHz to 8 ± 0.5 kHz. It also indicates that the signal processing algorithm of the HOC had great noise suppression performance in the aquaculture environment.

### 3.3. Recognition Performance of the r-Sigmoid Function

**Figure 6:**
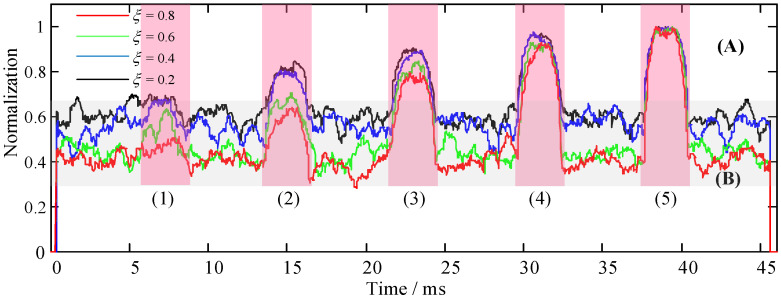
Recognition performance analysis with recognition coefficient *ξ*, where *λ* = 2. (**A**) Simulated target signal identification area. (**B**) Noise suppression area. (**1**–**5**) The SRCs of the simulated target signals with different energy ratios.

**Figure 7:**
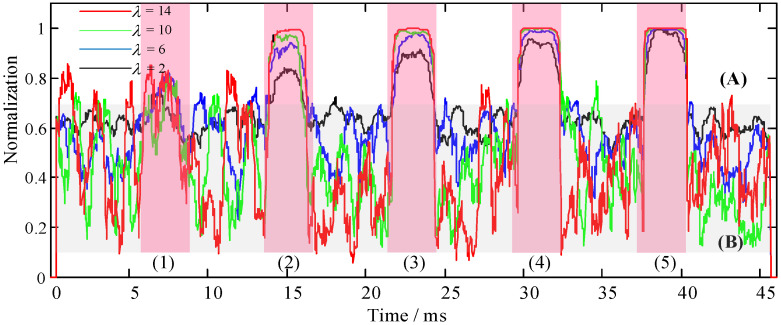
Recognition performance analysis of recognition coefficient *λ*, where *ξ* = 0.2. (**A**) The simulated target signal identification area. (**B**) Noise suppression area. (**1**–**5**) The SRCs of the simulated target signals with different energy ratios.

*λ* and *ξ* are the recognition coefficients of the recognition function, as shown in Equation (9). Corresponding recognition coefficients need to be set under different application scenarios or target signals, which also determines the performance of the recognition function for the HOC. In the simulation experiment, the signal (SNR = 10 dB) in Figure 4(4) was used as the sample signal to simulate the target signal with low SNR in a harsh environment. The effect of *λ* and *ξ* was singly analyzed through variable control, as was the performance of the recognition function. First, we set a fixed value of *λ* = 2, and the values of *ξ* were 0.2, 0.4, 0.6, and 0.8. We ran the algorithm program and drew the SRCs, as shown in Figure 6. Then, we set a fixed value of *ξ* = 0.2, and the values of *λ* were 2, 6, 10, and 14. We ran the algorithm program and drew the SRCs, as shown in Figure 7.

It can be seen from Figure 6 that the colors of the overall curve were red (*ξ* = 0.8), green (*ξ* = 0.6), blue (*ξ* = 0.4), and black (*ξ* = 0.2) from bottom to top, which indicates that the variation of *ξ* can inhibit noise (area Figure 6B). The larger the value of *ξ*, the more significant the noise suppression is, and the more stable it becomes. Nevertheless, by comparing the recognition function curves of the simulation target signal at five times, it can be intuitively seen that the target signal is completely submerged in the background noise and cannot be effectively distinguished in Figure 6(1) due to the minimum energy. From Figure 6(2–5), with the increase in the target signal energy, the performance effect of the signal recognition increases gradually; however, the signal recognition performance from left to right was less affected by the recognition coefficient *ξ* (Figure 6A).

Figure 7 shows that the curve fluctuation of the noise signal was severe (area Figure 7B), and the colors showed red (*λ* = 14), green (*λ* = 10), blue (*λ* = 6), and black (*λ* = 2) from bottom to top, whereas the target signal was relatively stable. This also indicates that the larger the recognition coefficient *λ* is, the better the recognition performance of the target signal (area Figure 7A). Furthermore, the energy of the weak signal was well balanced, whereas the contribution to noise suppression was very small (area Figure 7B). The relationship between the target signal energy and the performance effect of the signal recognition at five times was similar to that in Figure 6 (with the increase in signal energy, the performance effect of signal recognition also gradually increased). However, with the increase in the target signal energy, the signal recognition from left to right was less affected by the recognition coefficient *λ* (area Figure 7A).

According to the analyses in Figure 6 and Figure 7, the recognition coefficient *ξ* better suppressed the noise, but the target signal was also affected. The recognition coefficient *λ* better balances the weak target signal, but the noise suppression performance was not good. The two recognition coefficients balanced each other’s performance, making the recognition function better adapted to the application requirements or scenarios.

### 3.4. Algorithm Performance Analysis

**Figure 8:**
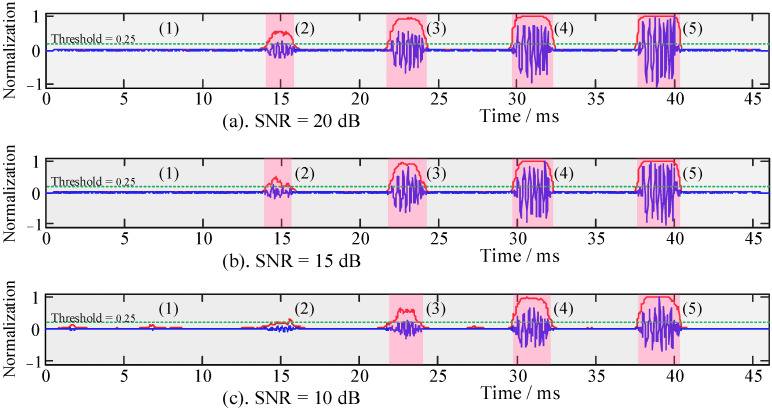
The SRCs of simulated signals with different SNRs. (**1**–**5**) The SRCs of the simulated target signals with different energy ratios.

The algorithm flow first calculates the HOC of different SNRs and then combines the recognition function to calculate the SRCs and identify the simulation target signals. According to Equation (10), and in combination with the analysis in Section 3.3, the values of the recognition coefficients were *λ* = 10 and *ξ* = 0.6. The SRCs were calculated with SNRs of 20 dB, 15 dB, and 10 dB. The threshold value of the SRC was set to 0.25 (green dotted line in Figure 8). The signal recognition processing results are shown in Figure 8, and the pink area shows a recognition area bigger than the recognition threshold of 0.25, and the gray area shows a recognition area smaller than the recognition threshold of 0.25.

As can be seen from Figure 8, when the SNRs were 10 dB and 15 dB, the recognition ability of the SRCs for the Figure 8(2) signal was insufficient, and only the Figure 8(3–5) signals could be recognized. When the SNR was 20 dB, the Figure 8(2) signal with small energy could be recognized; that is, the Figure 8(2–5) signals can be recognized. It indicates that when the SNR is low, the SRC has a weak ability to identify and distinguish the target signal. Meanwhile, the recognition resolution ability was gradually enhanced with an increase in SNR. In Figure 8, the recognition threshold of the pink areas is greater than 0.25, and the other gray areas are less than 0.25, which is the threshold decision after signal recognition to realize the classification process of useful and useless signals.

Furthermore, the cross-correlation coefficient was used to analyze the influence of the SNR change on the performance of the SRC algorithm, and the variation range of SNR was 0~20 dB. The cross-correlation coefficient can be formulated as follows in Equation (11):

(11) $$\overset{}{r}=\frac{\sum_{i=1}^{n}\left[(X_{i}-\bar{X})(Y_{i}-\bar{Y})\right]}{\sqrt{\sum_{i=1}^{n}{\left(X_{i}-\bar{X}\right)}^{2}}\sqrt{\sum_{i=1}^{n}{\left(Y_{i}-\bar{Y}\right)}^{2}}}$$

where X and Y denote the signal containing noise and the reference signal, respectively. Hence, the algorithm performance measurement index can be calculated as:

(12) $$\eta {=}_{}|\frac{r_{2}-r_{1}}{r_{2}}|\times 100\%$$

**Figure 9:**
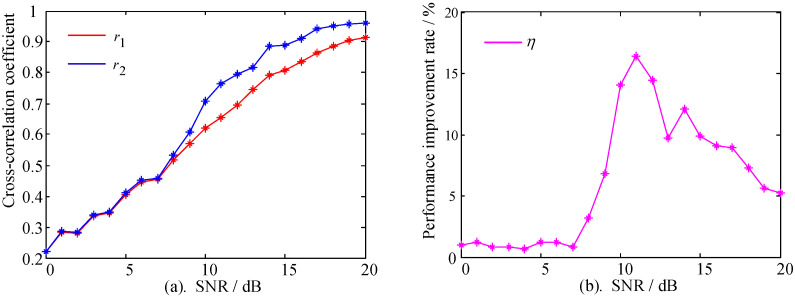
Comparison of the algorithm performance at different SNRs. (**a**) The cross-correlation coefficient curve comparison. (**b**) The SRC algorithm performance index curve.

The cross-correlation coefficient between the signal containing noise and the reference signal is $r_{1}$ (X can be referred to the signal in Figure 4b–d). The cross-correlation coefficient between the signal processed by the SRC algorithm and the reference signal is $r_{2}$ (X can be referred to the signal in Figure 8a–c), whereas *η* represents the algorithm performance index. The calculation results are shown in Figure 9.

Figure 9a shows that the cross-correlation coefficients $r_{1}$ and $r_{2}$ generally had an upward trend when the SNR changed from 0 to 20 dB. However, the performance improvements of $r_{2}$ were not monotonic. In Figure 9b, the performance improvements were measured by *η*, showing three stages of change. Within the SNR range of 0~7 dB, the change of *η* was gentle, with an average value of 0.977%, and the difference between $r_{1}$ and $r_{2}$ was not significant. When the SNR was within the range of 7~11 dB, the variation of *η* increased sharply. At the SNR of 11 dB, *η* reached the maximum of 16.44%, and the algorithm performance reached the optimal value of this condition, with the cross-correlation coefficient improved from $r_{1}$ = 0.623 to $r_{2}$ = 0.818. The change was moderate when the SNR was within the range of 11~20 dB, whereas the algorithm still had a good effect. In general, the algorithm maintained good and stable performance for short-term signal processing.

### 3.5. Processing and Verification of Algorithm Application

**Figure 10:**
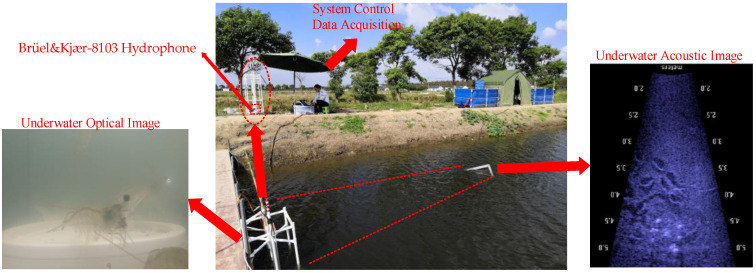
Layout of field verification experimental equipment.

Section 3.1, Section 3.2, Section 3.3 and Section 3.4 are the simulation experiments of the algorithm performance analysis. In order to further verify the recognition effectiveness of the SRC algorithm, the acoustic signals of *Penaeus vannamei* under the aquaculture engineering were observed using a hydrophone. The verification experiment site is located in the *Penaeus vannamei* breeding base in Fengxian District, Shanghai, and the layout of the experimental verification equipment is shown in Figure 10 below.

**Figure 11:**
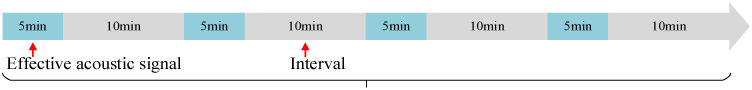
The distribution of data collection time in a day.

The hydrophone in the verification experiment was Type Brüel&Kjær-8103, a small transducer with high sensitivity. The receiving sensitivity of the hydrophone was −211 dB re 1 V/μPa. The hydrophone was placed at 1.5 m depth underwater. At the same time, underwater optical images and underwater acoustic images were applied as auxiliary analyses. The signal’s sampling frequency was 48 kHz, the data width was 24 bit, and the distribution of data collection time in a day (20 min/day), as shown in Figure 11 below. The total length of the acquisition signal time was 60 min (20 min/day × 3 days). The acquired signal segment (200 ms) was used as a sample signal to display the algorithm effect.

**Figure 12:**
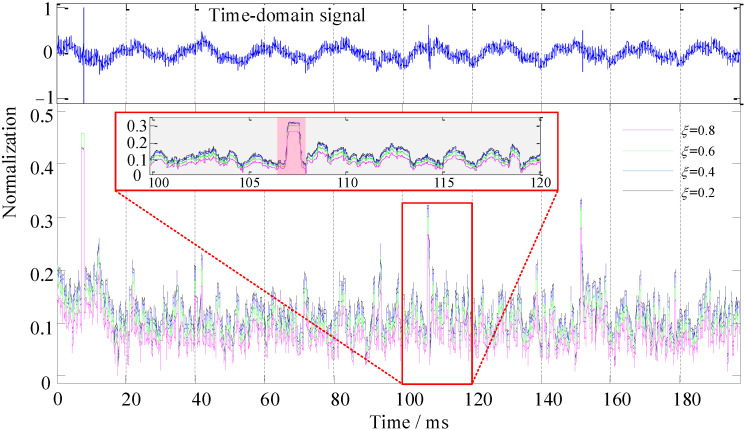
Recognition performance of *ξ* in the aquaculture engineering application experiment, where *λ* = 2.

**Figure 13:**
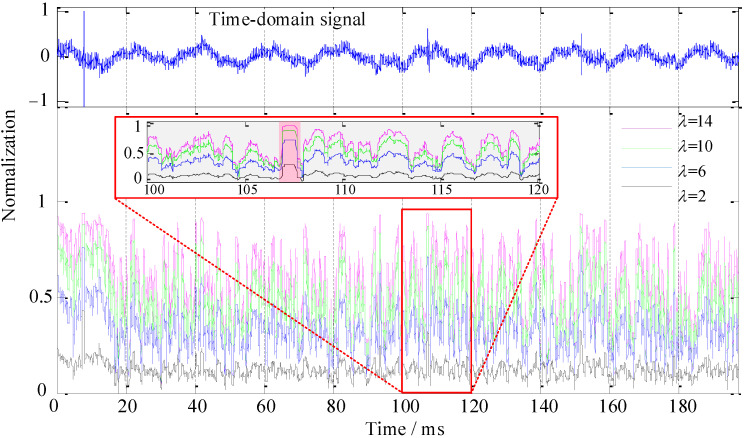
Recognition performance of *λ* in the aquaculture engineering application experiment, where *ξ* = 0.2.

Next, we discuss the effect of the recognition coefficients *λ* and *ξ* in the sample signal (refer to Section 3.3 for specific methods). The results of the algorithm processing are shown in Figure 12 and Figure 13.

In Figure 12, the overall SRCs represent the stratification phenomenon, and they are purple (*ξ* = 0.8), green (*ξ* = 0.6), blue (*ξ* = 0.4), and black (*ξ* = 0.2) from bottom to top, which relatively intuitively illustrate that the recognition coefficient *ξ* had a good effect on noise suppression. As seen from the magnified area of Figure 12, the SRC of the noise part was less than 0.2 (grey area), whereas the SRC of the target signal was 0.25 to 0.35 (pink area).

**Figure 14:**
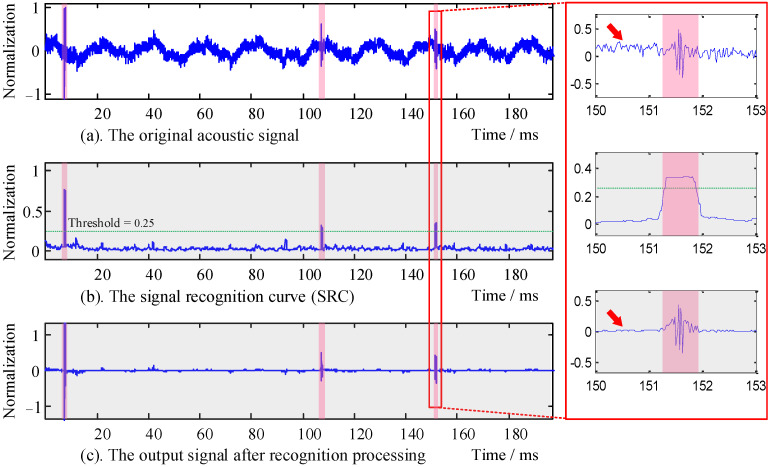
The SRC algorithm processing effect of the *Penaeus vannamei* acoustic signal.

In Figure 13, the overall SRCs show stratification with severe fluctuation. Meanwhile, the colors of the overall curve show purple (*λ* = 2), green (*λ* = 6), blue (*λ* = 10), and black (*λ* = 14) from top to bottom. From the magnified area of Figure 13, the recognition coefficient *λ* can compensate for the overall signal energy, but the noise energy was amplified simultaneously. Therefore, by combining the performance effects of the recognition coefficients *ξ* and *λ*, the recognition function could simultaneously suppress the noise and compensate the energy of the target signal. In the signal processing of the aquaculture engineering application experiment, the recognition coefficients were selected as *ξ* = 0.8 and *λ* = 6, as shown in Figure 14.

The original acoustic signals of *Penaeus vannamei* collected by the hydrophone are shown in Figure 14a. There was both low-frequency and high-frequency interference in the collected signal time-domain waveform. The collected signal was processed by the HOC and r-sigmoid recognition function, and the SRC of the signal is shown in Figure 14b. The output signal after recognition processing is shown in Figure 14c. From the analysis of the local amplification, it can be seen that the target signal duration was about 1 ms, and the algorithm had a strong recognition ability for the target signal. The red arrow indicates that the algorithm had a good inhibition effect on the environmental background noise.

## 4. Conclusions

This paper proposes an SRC algorithm based on bioacoustic hydrophone signals. The main idea is the joint processing of the HOC and the r-sigmoid function. Combining these two aspects can improve the recognition and detection performance of non-stationary pulse signals in an underwater background noise environment. The processing results of the simulation experiment and the application case show that the SRC algorithm has a good effect on the noise suppression and detection of underwater target signals. The analysis found that when the SNR is greater than 7 dB, the SRC algorithm is effective, and the performance improvement is maximized when the SNR is 11 dB. The SRC algorithm can effectively suppress the noise through *ξ*, and *λ* can effectively compensate the energy of the target signal. Under the joint action of the two coefficients, the algorithm can effectively achieve underwater background noise reduction and target signal energy balance. It is beneficial to target signal recognition and information extraction. This method can help signal monitoring and research of *Penaeus vannamei* in aquaculture. The follow-up work will further improve the robustness of the SRC algorithm and try to popularize and apply it in the field of smart aquaculture engineering.

## Data Availability

Data are available, please contact the authors by e-mail.
